# Anemia and Bone Marrow Suppression After Intra-Arterial Chemotherapy in Children With Retinoblastoma: A Retrospective Analysis

**DOI:** 10.3389/fonc.2022.848877

**Published:** 2022-07-25

**Authors:** Changjuan Zeng, Minglei Han, Jiayan Fan, Xiaoyu He, Renbing Jia, Li Li, Xuyang Wen, Xuefei Song, Lili Hou

**Affiliations:** ^1^ Shanghai Jiao Tong University School of Nursing, Shanghai, China; ^2^ Department of Ophthalmology, Ninth People's Hospital, Shanghai Jiao Tong University School of Medicine, Shanghai, China; ^3^ Shanghai Key Laboratory of Orbital Diseases and Ocular Oncology, Shanghai, China; ^4^ Department of Nursing, Ninth People's Hospital, Shanghai Jiao Tong University School of Medicine, Shanghai, China; ^5^ Department of Nursing, Mental Health Center, Shanghai Jiao Tong University School of Medicine, Shanghai, China

**Keywords:** retinoblastoma, anemia, bone marrow suppression, intra-arterial chemotherapy, multivariate regression

## Abstract

**Background:**

Retinoblastoma (Rb) is a common ocular malignant tumor in children. Intra-arterial chemotherapy (IAC) has been widely used in children with Rb and has achieved an ideal therapeutic effect. However, IAC has side effects, including anemia and bone marrow suppression, for which explicit evidence on the risk factors is lacking. This study aimed to evaluate the covariates that may affect the occurrence of anemia and bone marrow suppression in children with Rb after IAC.

**Methods:**

Children with Rb admitted between May 2019 and January 2021 were included into the study. The differences in the number of children with anemia and bone marrow suppression before and after IAC according to different covariates were recorded. All potential impact factors were included into the univariate and multivariate regression models to identify the related covariates of post-IAC anemia and bone marrow suppression.

**Results:**

Data of 282 children with Rb were retrospectively collected. After IAC, children with Rb had increased severities of anemia (p <0.0001, chi-square test) and bone marrow suppression (p = 0.001, chi-square test). Moreover, the number of children with Rb who had an increased cross-level change in the severity of anemia and degree of bone marrow suppression was 80 (41.24%) and 64 (32.49%), respectively. The univariate regression analysis showed that numerous factors (such as pre-IAC intravenous chemotherapy, results of pre-IAC routine blood tests, and some serological indicators for liver and kidney function) affected the anemia severity and degree of bone marrow suppression in children with Rb after IAC. Additionally, the predictive model of the multivariate regression could predict anemia and bone marrow suppression.

**Conclusion:**

Children with Rb may have an increased risk of anemia and bone marrow suppression after IAC, but this is temporary and can be influenced by several factors. Therefore, IAC should be maintained as the standard of care. We generated predictive equations for predicting anemia severity and degree of bone marrow suppression, which can guide the prediction and timely control of anemia and bone marrow suppression after IAC.

## Introduction

Retinoblastoma (Rb) is a common ocular malignant tumor in children. Rb occurs in 1 out of every 15,000–20,000 newborns and accounts for 4% of all malignant tumors in children (1). If the tumor is confined within the eye, it can be controlled by enucleation (2). If the tumor spreads to the entire eyeball or body, the survival rate is greatly reduced (3, 4). The 1-, 3-, and 5-year overall survival rates of patients with retinoblastoma are 95%, 86%, and 83%, respectively (5).

Rb treatment has gradually changed from enucleation and radiotherapy to chemoreduction and local treatment (6, 7). Chemotherapy can effectively reduce the tumor volume and create favorable conditions for local surgical treatment and is currently one of the primary treatment methods for Rb in children (8). It is divided into systemic intravenous chemotherapy and intra-arterial chemotherapy (IAC) (9, 10). Intravenous chemotherapy (IVC) is a primary treatment method for bilateral cases and is the only available treatment in low-income countries. As a postoperative adjuvant treatment, IVC can largely prevent distant metastasis in patients with high-risk pathological factors after eyeball enucleation (9, 10). However, the blood–retinal barrier can limit the effect of drugs on the tumor site, resulting in unsatisfactory chemotherapy effects and an increased risk of systemic side effects. Unlike in intravenous chemotherapy, in IAC, drugs are delivered through the arterial system under digital subtraction angiography and are delivered directly into the tumor *via* the ophthalmic artery (11). This increases the drug concentration in the tumor tissue, while reducing it in normal tissues, thereby reducing systemic side effects (12).

Previous studies have shown that patients with Rb have varying severities of anemia and degrees of myelosuppression after IAC, but there is no explicit evidence of their risk factors (13, 14). Therefore, it is difficult to intervene before IAC in anticipation of IAC-related complications. Our study aimed to comprehensively evaluate the covariates and risk factors of anemia and bone marrow suppression in children with Rb after IAC.

## Materials and Methods

All the patients in this study were from the Department of Ophthalmology, Ninth People’s Hospital, Shanghai Jiao Tong University School of Medicine. The patients’ treatment information, including surgical records, was collected from the electronic medical record system. The doctors who performed interventional treatments on these children had extensive clinical experience. The patients’ medical information, including medication status, intervention process, and serological examination reports of patients, were stored in an encrypted public network disk and was accessible only to the clinician who processed the data. This study was approved by the Ninth people’s hospital, Shanghai Jiao Tong University School of Medicine (SH9H-2019-T291-3), and the need for informed consent was waived, owing to the retrospective nature of the study.

### Study Design

We performed a retrospective study and used a multivariate regression analysis to summarize the clinical data and assess the risk of anemia and bone marrow suppression in children with Rb after IAC.

### Study Subjects

Children with Rb treated with IAC between May 2019 and January 2021 were enrolled in this study.

**Inclusion criteria:**

1) Clinical diagnosis of Rb.
2) Children who met the indications for IAC and were treated accordingly.
3) No history of central system diseases, such as brain metastasis or epilepsy.

**Exclusion criteria:**

1) Children with severe systemic diseases.
2) Children with iodine contrast agent allergy.
3) Children suffering from systemic blood diseases.
4) Children with severe anemia [hemoglobin (Hb) level ≤60 g/L] or bone marrow suppression [white blood cell (WBC) count <2.0 × 10^9^/L, Hb <80 g/L, and platelet (PLT) count <50 × 10^9^/L] before IAC.

### Treatment Regimens

After anesthesia was administered, the surgical site was disinfected, and the femoral artery on the affected side was punctured using the Seldinger technique. We then inserted the vascular sheath and injected heparin. Under radiographic guidance, a Cobra catheter was inserted into the internal carotid artery on the affected side. Thereafter, the intensifier was turned to an angle of 90°, and the head image was captured in a lateral position. A contrast agent for digital subtraction angiography of the internal carotid artery was administered manually. After identifying the ophthalmic artery, the ev3 micro-guide wire was used to guide the 1.7F ev3 45° micro-catheter for ophthalmic artery intubation. After the angiography was performed, intra-ophthalmic arterial chemotherapy was administered. The IAC regimen was a 2- or 3-drug regimen (melphalan, topotecan, carboplatin, or a combination thereof); 3–4 cycles were administered. The dose of chemotherapeutic drugs increased with age and tumor size (melphalan, 3.5–7.5 mg; topotecan, 1 mg; and carboplatin, 20 mg) (15). When intubation of the ophthalmic artery was not feasible, the route of administration was the main primary ophthalmic artery (OA) pathway or the external carotid artery (ECA) pathway. The alternative ECA pathway does not affect IAC efficacy in Rb treatment (16). The sheath was removed postoperatively.

### Covariates

The data retrieved from the medical charts included sex, age, duration of Rb, affected eyes (left, right, or bilateral), International Intraocular Retinoblastoma Classification group at diagnosis, times of IAC, chemotherapy drugs (melphalan + topotecan, melphalan + carboplatin), duration of surgery, duration of heparin administration, height, weight, head circumference, chest circumference, operating doctors’ details, and whether chemotherapy (intravenous chemotherapy or arterial chemotherapy) was administered 6 months before this investigation

### Analysis of Anemia and Bone Marrow Suppression in Children With Rb Before and After IAC

Children with Rb were divided into the no anemia (Hb, >120 g/L), mild anemia (Hb, 91–120 g/L), moderate anemia (Hb, 61–90 g/L), severe anemia (Hb, 31–60 g/L), and extreme anemia (Hb, <30 g/L) groups according to the Hb levels. The difference in anemia severity before and after IAC was compared.

The degree of bone marrow suppression was evaluated according to the grading standard of acute and subacute toxicity of anti-cancer drugs of the World Health Organization. According to the Hb, WBC, and PLT levels during routine blood tests, the degree of bone marrow suppression was divided into grade 0 (WBC >4.0 × 10^9^/L, Hb >110 g/L, or PLT ≥100 × 10^9^/L), grade I (3.0< WBC ≤4.0 × 10^9^/L, 95< Hb ≤110 g/L, or 75< PLT ≤99 × 10^9^/L), grade II (2.0< WBC ≤3.0 × 10^9^/L, 80< Hb ≤95 g/L, or 50< PLT ≤75 × 10^9^/L), grade III (1.0< WBC ≤2.0 × 10^9^/L, 65< Hb ≤80 g/L, or 25< PLT ≤50 × 10^9^/L), and grade IV (WBC ≤1.0 × 10^9^/L, Hb ≤65 g/L, or PLT ≤25 × 10^9^/L). We compared the changes in WBC, Hb, and PLT levels and graded the changes in bone marrow suppression before and after IAC.

### Univariate and Multivariate Regression Analysis of Anemia and Bone Marrow Suppression Before and After IAC in Children With Rb

To fully understand the factors that affect the occurrence of anemia and bone marrow suppression in children with Rb after IAC, we incorporated all covariates and other factors that may affect the results (such as routine blood tests, liver and kidney function, and arterial or intravenous chemotherapy before IAC) into the univariate regression model to identify the most relevant variables. After analyzing the results of the univariate regression model, variables with a significant effect on anemia and bone marrow suppression were included in the multivariate regression analysis for further exploration and verification.

### Constructed Predictive Model on Anemia and Bone Marrow Suppression After IAC

According to the results of the multivariate regression analysis, we generated formulas to predict the possible status of anemia or bone marrow suppression after IAC. The generated equations were all based on the multivariate regression analysis results.

### Statistical Analysis

All statistical analyses were performed using R 4.0.2 software. The chi-square test was used to compare the distribution of categorical variables (such as sex, age group) between the groups. The t-test or Mann–Whitney test was used to compare differences in continuous variables (such as age and duration of surgery) according to the data distribution. Univariate and multivariate regression models were constructed to explore the association of demographic and clinicopathological characteristics with anemia and bone marrow suppression after IAC. The grade of anemia and bone marrow suppression were used as dependent variables, and covariates were used as independent variables. Variables with a two-tailed p-value <0.1 in the univariate analysis were analyzed using the multivariate regression model. A p-value <0.05 was considered statistically significant.

## Results

### Study Population

**Figure 1:**
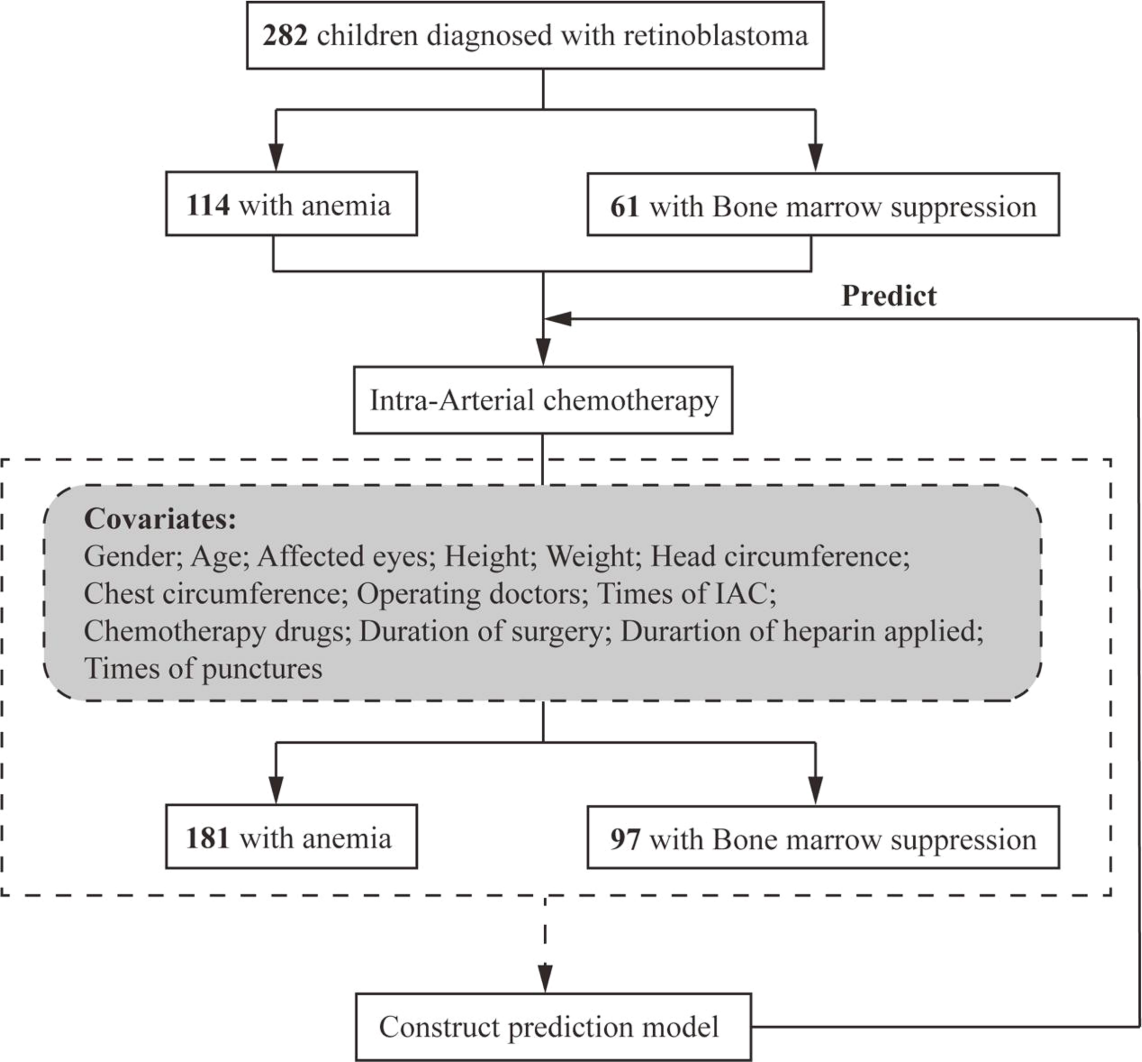
Study population.

The number of patients who received melphalan, melphalan + topotecan, melphalan + carboplatin, and melphalan + topotecan + carboplatin as chemotherapy drugs was 3, 163, 119 and 1, respectively, accounting for a total of 286 patients. Since the number of patients who used melphalan and melphalan +topotecan +carboplatin as chemotherapy drugs was too small, we excluded the 4 patients to avoid increasing analysis bias. Therefore, the final number of patients enrolled into our study were 282.The data of 282 children with Rb treated with IAC were analyzed in this study. Among them, there were 114 (40.43%) and 181 (64.18%) children with anemia before and after IAC, respectively, and 61 (21.63%) and 97 (34.40%) children with bone marrow suppression before and after IAC (**Figure 1**), respectively. **Table 1** summarizes the distribution of anemia and bone marrow suppression according to demographics or clinical data. There were no significant differences in these variables between the patients, except for the time of operation and heparin use in terms of possible covariates of bone marrow suppression.

**Table 1:** Anemia and bone marrow suppression in children with Rb before and after IAC.

	Total patients (N = 282)					
	Anemia			Bone marrow suppression		
	Before IAC (N = 114)	After IAC (N = 181)	p value	Before IAC (N = 61)	After IAC (N = 97)	p value^#^
	Number (percentage)			Number (percentage)		
**Sex**			0.5674			0.947
Male	61 (53.51)	103 (56.91)		33 (54.10)	53 (54.64)	
Female	53 (46.49)	78 (43.09)		28 (45.90)	44 (45.36)	
**Age [mean (SD)]^&^**	3.52 (1.23)	3.77 (1.26)	0.0949	3.45 (1.52)	3.84 (1.18)	0.0781
**Laterality at diagnosis**			0.4062			0.5092
Left	68 (69.65)	98 (54.14)		34 (55.74)	51 (52.58)	
Right	41 (35.96)	78 (43.09)		24 (39.34)	44 (45.36)	
Bilateral	5 (4.39)	5 (2.76)		3 (4.92)	2 (2.06)	
**Times of IAC**			0.6479			0.7987
1	34 (29.82)	49 (27.07)		14 (22.95)	27 (27.84)	
2	31 (27.19)	49 (27.07)		20 (32.79)	25 (25.77)	
3	25 (21.93)	47 (25.97)		13 (21.31)	26 (26.80)	
4	18 (15.79)	21 (11.60)		9 (14.75)	12 (12.37)	
≥5	6 (5.26)	15 (8.29)		5 (8.20)	7 (7.21)	
**Chemotherapy drugs**		0.7946			0.3257
Melphalan + Topotecan	66 (57.89)	102 (56.35)		40 (65.57)	56 (57.73)	
Melphalan + carboplatin	48 (42.11)	79 (43.65)		21 (34.43)	41 (42.27)	
**Duration of surgery (min) [mean (SD)]^&^**	70.83 (23.31)	71.7 (19.69)	0.7328	64.30 (16.59)	72.28 (19.90)	**0.0099**
**Duration of heparin applied (min) [mean (SD)]^&^**	66.02 (20.46)	66.74 (16.72)	0.7411	60.74 (13.13)	67.32 (16.75)	**0.0102**
**Height (cm) [mean (SD)]^&^**	86.46 (11.20)	88.29 (11.38)	0.181	87.41 (14.35)	88.67 (10.98)	0.5365
**Weight (kg) [mean (SD)]^&^**	12.51 (2.94)	13.02 (3.10)	0.1631	12.91 (3.94)	13.04 (3.01)	0.8158
**Head circumference (cm) [mean (SD)]^&^**	47.04 (2.84)	47.43 (2.63)	0.2647	47.14 (3.22)	47.55 (2.44)	0.4034
**Chest circumference (cm) [mean (SD)]^&^**	50.52 (4.09)	51.36 (4.01)	0.1058	51.86 (5.23)	51.68 (4.39)	0.786
**Operating doctors**		0.8376			0.7208
Dr. one	87 (76.32)	140 (77.35)		45 (73.77)	74 (76.29)	
Dr. two	27 (23.68)	41 (22.65)		16 (26.23)	23 (23.71)	

^#^The p value was calculated by the chi-square test.

^&^The p value was calculated by the t test or Mann-Whitney test.

The meaning of the bold values are p-value <0.05, which was considered statistically significant.

### Anemia Severity and Bone Marrow Suppression Degree Were Enhanced After IAC in Children With Rb

**Figure 2:**
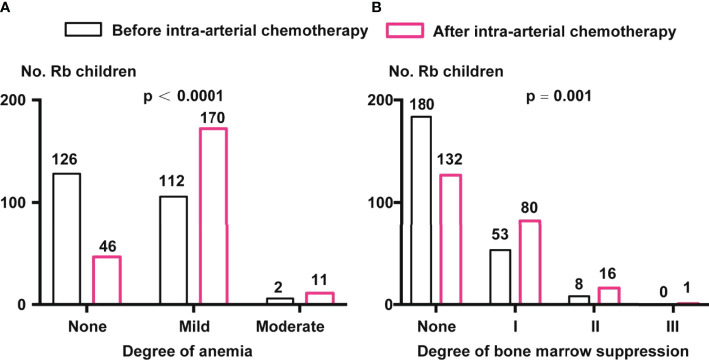
Comparison of anemia and bone marrow suppression before and after IAC. **(A)** Anemia classification before and after IAC. Percentage of children with Rb in mild and moderate anemia were enhanced after IAC (p < 0.0001 by chi-square test). **(B)** Degree of bone marrow suppression before and after IAC. Percentage of children with Rb in I, II, and III grade of bone marrow suppression were enhanced after IAC (p = 0.001 by chi-square test).

The data of approximately 19.5% of the patients were missing; these patients were excluded. We found that there were 126 (52.50%), 112 (46.67%), and 2 (0.83%) children with Rb with no, mild, and moderate anemia, respectively. After IAC, the proportion of children with mild and moderate anemia increased significantly, with 170 children having mild anemia (74.89%) and 11 having moderate anemia (4.85%) (p <0.0001, chi-square test) (**Figure 2A**).

Before IAC, there were 180 (74.69%), 53 (21.99%), and 8 (3.32%) children with grades 0, I, and II bone marrow suppression, respectively. After IAC, the proportion of children with grades I (80, 34.93%), II (16, 6.99%), and III (1, 0.44%) (p = 0.001, chi-square test) (**Figure 2B**).

### Cross-Level Changes in Anemia Severity and Bone Marrow Suppression Degree After IAC in Children With Rb

**Figure 3:**
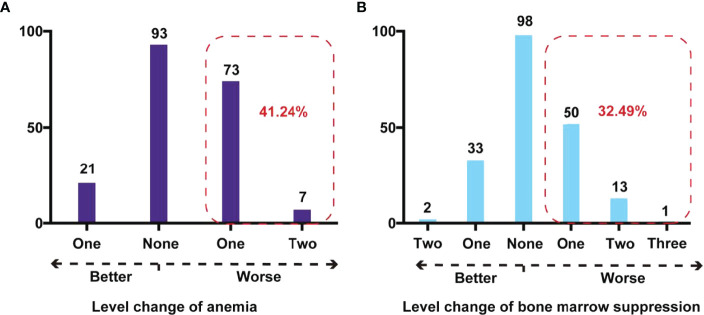
Cross-level impact on degree of anemia and bone marrow suppression after IAC in children with Rb. **(A)** Cross-level change of anemia in Rb children. The red dashed box showed the number and percentage of children with Rb whose anemia stage deteriorated after IAC. **(B)** Cross-level change of bone marrow suppression in children with Rb. The red dashed box showed the number and percentage of children with Rb whose bone marrow suppression stage deteriorated after IAC.

Regarding “anemia severity,” there were 93 (47.94%) children whose classification remained unchanged. After IAC, there were 73 (37.63%) and 7 (3.61%) children with Rb who had a cross-level change in anemia severity to one and two levels up, respectively (**Figure 3A**).

Regarding the “degree of bone marrow suppression,” 98 children (49.75%) did not show a change in the degree before and after IAC. However, there were 50 (25.38%), 13 (6.60%), and 1 (0.51%) children with Rb who had a cross-level increase in the degree of bone marrow suppression by one, two, and three grades, respectively (**Figure 3B**).After IAC, the proportion of anemia and bone marrow suppression in children with Rb who developed worse conditions were 41.24% and 32.49%.

### Univariate Regression Analysis of IAC Related to Anemia Severity and Degree of Bone Marrow Suppression

To clarify possible reasons that affect the cross-level changes in anemia severity and bone marrow suppression degree, we collected and analyzed available information from the patients with detailed records (such as results of routine blood and liver and kidney function tests before and after IAC). After excluding the patients with incomplete data, 194 and 197 participants were enrolled in the univariate regression analysis of anemia and bone marrow suppression, respectively.

In the univariate regression model generated by taking the cross-level changes in anemia severity and degree of bone marrow suppression before and after IAC as the dependent variables, and covariates as the control variables, the factors that affected the change in the anemia severity and the degree of bone marrow suppression included anemia severity before IAC, degree of bone marrow suppression before IAC, intravenous and arterial chemotherapy within 1–2 months before IAC, WBC count, red blood cell count, Hb, and PLT count before IAC, mean erythrocyte Hb concentration, red cell volume distribution width, lymphocyte count (absolute), hematocrit, neutrophil count (absolute), alanine aminotransferase, aspartate aminotransferase, serum total bilirubin and serum direct bilirubin (**Table 2**). In addition, the covariates that did not show statistically significant effects on the cross-level changes in anemia severity and degree of bone marrow suppression are listed in the **Supplementary file (S Table 1)**.

**Table 2:** Univariate regression analysis of IAC related to anemia severity and degree of bone marrow suppression.

	Total patients (N = 282)								
	Anemia (N = 194)				Bone marrow suppression (N = 197)				
	Worse (N = 80)	Unchanged (N = 93)	Better (N = 21)	p value^#^	Worse (N = 64)		Unchanged (N = 98)	Better (N = 35)	P value^#^
**Anemia classification before IAC**				<0.001					<0.001
none	78 (97.5)	23 (24.7)	0 (0.0)		43 (68.3)		51 (52.0)	8 (22.9)	
mild	2 (2.5)	70 (75.3)	20 (95.2)		20 (31.7)		47 (48.0)	26 (74.3)	
moderate	0 (0.0)	0 (0.0)	1 (4.8)		0 (0.0)		0 (0.0)	1 (2.9)	
**Degree of bone marrow suppression before IAC**				0.006					<0.001
none	72 (90.0)	61 (65.6)	15 (71.4)		62 (96.9)		88 (89.8)	0 (0.0)	
I	7 (8.8)	28 (30.1)	5 (23.8)		2 (3.1)		10 (10.2)	28 (80.0)	
II	1 (1.2)	4 (4.3)	1 (4.8)		0 (0.0)		0 (0.0)	7 (20.0)	
**Intravenous chemotherapy within 1-2 months before IAC**				0.042					0.01
None	69 (86.2)	73 (78.5)	13 (61.9)		58 (90.6)		77 (78.6)	23 (65.7)	
Yes	11 (13.8)	20 (21.5)	8 (38.1)		6 (9.4)		21 (21.4)	12 (34.3)	
**Arterial chemotherapy within 1-2 months before IAC**				0.706					0.033
None	47 (58.8)	50 (53.8)	13 (61.9)		28 (43.8)		63 (64.3)	21 (60.0)	
Yes	33 (41.2)	43 (46.2)	8 (38.1)		36 (56.2)		35 (35.7)	14 (40.0)	
**WBC count [median (IQR)]^&^**				0.208					<0.001
	6.16 [5.18, 7.62]	5.76 [4.40, 7.46]	5.45 [5.09, 6.48]		5.81 [4.96, 7.14]		6.49 [5.42, 7.85]	3.93 [3.50, 5.55]	
**RBC count [median (IQR)]^&^**				<0.001					<0.001
	4.61 [4.43, 4.82]	4.19 [3.98, 4.42]	4.09 [3.91, 4.27]		4.48 [4.14, 4.76]		4.41 [4.12, 4.62]	4.11 [3.71, 4.29]	
**Hemoglobin [median (IQR)]^&^**				<0.001					<0.001
	129.00 [124.00, 134.00]	116.00 [112.00, 120.00]	115.00 [111.00, 118.00]		124.60 [118.50, 133.00]	121.00 [115.25, 127.00]		109.00 [100.30, 118.00]	
**platelet count [median (IQR)]^&^**				<0.001					<0.001
	344.50 [294.50, 394.20]	220.00 [187.00, 244.00]	236.00 [195.00, 263.00]		304.00 [251.00, 375.25]		268.00 [229.75, 329.75]	187.00 [147.50, 244.50]	
**Hematocrit (HCT) [median (IQR)]^&^**				0.022					0.138
	0.46 [0.38, 37.73]	31.35 [0.35, 35.10]	33.30 [26.40, 34.40]		33.35 [0.38, 37.62]		32.40 [0.37, 36.00]	29.70 [0.37, 34.50]	
**Mean erythrocyte hemoglobin concentration [median (IQR)]^&^**				0.001					0.001
	340.00 [333.00, 346.00]	334.00 [327.00, 341.25]	336.00 [330.00, 341.00]		339.00 [332.50, 345.50]		336.00 [330.00, 343.00]	331.43 [324.50, 336.00]	
**Red Cell volume Distribution Width [median (IQR)]^&^**				0.006					<0.001
	14.50 [13.30, 16.00]	14.00 [13.40, 14.90]	13.10 [12.30, 14.63]		14.90 [14.00, 16.25]		13.90 [13.10, 14.80]	13.60 [13.10, 14.70]	
**Lymphocytes (absolute) [median (IQR)]^&^**				0.056					0.12
	3.02 [2.37, 4.43]	2.67 [2.06, 3.60]	3.02 [2.10, 3.60]		2.75 [1.78, 3.95]		3.10 [2.32, 4.04]	2.51 [2.11, 3.40]	
**Neutrophils (absolute) [median (IQR)]^&^**				0.025					0.447
	2.38 [1.70, 3.09]	2.10 [1.65, 2.80]	1.72 [1.40, 2.36]		2.20 [1.67, 2.87]		2.00 [1.42, 2.72]	2.10 [1.70, 2.98]	
**Alanine aminotransferase [median (IQR)]^&^**				0.017					0.353
	19.65 [15.00, 24.45]	16.00 [14.00, 19.00]	16.00 [14.00, 21.23]		18.00 [14.00, 23.00]		16.00 [13.80, 21.90]	16.50 [14.70, 19.00]	
**Aspartate aminotransferase [median (IQR)]^&^**				0.027					0.041
	36.50 [33.00, 41.58]	35.00 [30.00, 38.00]	35.50 [30.50, 38.00]		37.00 [33.45, 42.17]		35.00 [30.50, 38.55]	35.00 [30.25, 36.75]	
**Serum total bilirubin [median (IQR)]^&^**				0.072					0.118
	6.00 [4.90, 7.28]	7.00 [5.07, 8.93]	6.35 [5.00, 7.78]		5.90 [4.85, 7.03]		6.72 [5.00, 8.95]	6.00 [5.00, 7.78]	
**Serum direct bilirubin [median (IQR)]^&^**				0.3					0.01
	2.00 [1.00, 2.30]	2.00 [1.67, 2.99]	2.00 [1.50, 2.65]		1.70 [1.00, 2.10]		2.00 [1.52, 3.00]	2.00 [1.50, 2.02]	

^#^The p value was calculated by the chi-square test.

^&^The p value was calculated by the t test or Mann-Whitney test.

### Multivariate Regression Analysis and Predictive Model on Anemia Severity and Degree of Bone Marrow Suppression After IAC

After including all the variables that were identified the univariate regression analysis into the multivariate regression model, the factors affecting anemia severity in children with Rb after IAC were anemia severity and bone marrow suppression degree before IAC, red cell volume distribution width, PLT count, lymphocyte count (absolute), and neutrophil count (absolute). Meanwhile, the factors that affected the degree of bone marrow suppression were arterial chemotherapy within 1–2 months before IAC, red cell volume distribution width, and serum direct bilirubin (**Table 3**).

**Table 3:** Multivariate regression analysis of IAC on anemia severity and degree of bone marrow suppression.

		Est.	Std.E	Statistic	p value
**Anemia classification**	**Degree of anemia before IAC**	1.36	0.67	2.02	0.043
	**Degree of bone marrow suppression before IAC**	1.40	0.57	2.47	0.014
	**Red Cell volume Distribution Width**	0.69	0.18	3.74	0.000
	**platelet count**	0.01	0.01	2.35	0.019
	**Lymphocytes (absolute)**	-0.23	0.14	-1.66	0.098
	**Neutrophils (absolute)**	0.56	0.24	2.38	0.017
**Degree of bone marrow suppression**	**Arterial chemotherapy within 1-2 months before IAC**	0.770	0.363	2.120	0.034
	**Red Cell volume Distribution Width**	0.576	0.125	4.615	0.000
	**Serum direct bilirubin**	-0.463	0.201	-2.298	0.022

Based on the results of the multivariate regression model, we generated an equation to predict anemia and bone marrow suppression before IAC. The equations for predicting anemia severity and degree of bone marrow suppression were based on **Table 3**. The equations are shown below.

***Anemia severity after IAC =*** *−13.230 + 1.356 × (anemia severity before IAC) + 1.398 × (degree of bone marrow suppression before IAC) + 0.688 × (red cell volume distribution width) + 0.012 × (PLT count) − 0.230 × (absolute lymphocyte count) + 0.565 × (absolute neutrophil count)*

***Degree of bone marrow suppression after IAC =*** *−8.251 + 0.770 × (arterial chemotherapy within 1–2 months before IAC) + 0.576 × (red cell volume distribution width) − 0.463 × (serum direct bilirubin)*

## Discussion

Although anemia and myelosuppression occur in most patients with cancer receiving chemotherapy, so far, no clinically useful tool for the accurate identification of patients at risk of anemia and myelosuppression has been developed. This study collected information on children with Rb before and after IAC and constructed multivariate regression models to initially explore the factors that may affect anemia and bone marrow suppression after IAC, evaluate the risk of anemia or bone marrow suppression for children with Rb before IAC, and provide clinical guidance for surgeons.

Rb is a devastating, blinding, and life-threatening disease in children; 36%, 77%, 95%, and 100% of patients with Rb who abandon treatment die within 12, 24, 36, and 48 months after diagnosis, respectively (17). Since IAC was first introduced in China in 2009, it has been used widely in some retinoblastoma treatment centers (18). IAC has become a very effective treatment option for Rb. By delivering chemotherapeutic drugs directly to the tumor, IAC can ensure that the drug concentration in the tumor is significantly increased while the peripheral blood chemotherapeutic drug concentration is low (19). Therefore, IAC can maximize the tumoricidal effect of chemotherapy drugs and reduce their adverse effects (20, 21). Thus far, IAC has a high rate of ocular salvage and a low incidence of serious complications in patients with refractory Rb (22). Melphalan-based IAC is an innovative treatment for patients with Rb because of its related high globe salvage rate. The overall globe salvagerate of patients receiving IAC as the main treatment is 79.6% (23), and the technical success rate is 98.5% (24). IAC has gradually been first-line treatment for unilateral retinoblastoma and very effective treatment for refractory retinoblastoma.

Although IAC has good clinical treatment effects in children with Rb, it still has adverse effects on the eyes and systemic effects (19). A systematic review of the application of IAC in refractory Rb showed that the commonest systemic complications were nausea/vomiting (20.5%), neutropenia (14.1%), fever (8.2%), and bronchospasm (6.2%) (22).The commonest ocular complications were retinopathy (32%) and eyelid edema (29.7%) (23). Ocular complications (ophthalmic artery obstruction, vitreous hemorrhage, and optic neuropathy) and systemic complications (transient pancytopenia, ototoxicity, and leukemia) have been associated with shield use (25). The local side effects of IAC are a concern for many scholars. Although the systemic side effects caused by IAC are not serious, they affect the quality of life of children and should not be ignored, especially anemia and bone marrow suppression caused by IAC. Although the human body tries to counteract the effects of anemia through various mechanisms, almost every organ system of the human body will eventually be affected. Patients experience a variety of symptoms, ranging from skin chills, heart failure, and palpitations to pulmonary edema, dizziness, depression, and severe cognitive impairment. Anemia seriously affects the quality of life of patients (26). Anemia was significantly associated with poor performance status (27). The incidence of bone marrow suppression after IAC in infants less than 3 months old with advanced Rb was 7.7% (18). The European Cancer Anaemia Survey evaluated anemia in 15,367 patients with cancer in Europe for 6 months and found that the incidence of anemia was 53.7% (Hb <10.0 g/dL) (27). Anemia and bone marrow suppression is very common in patients with cancer, and their pathogenesis is complex.

Our study found that children with Rb had varying anemia severities and bone marrow suppression degrees after IAC. This study investigated 282 retinoblastoma cases; the rates of post-IAC anemia and myelosuppression in children were 64.18% and 34.40%, respectively, which were significantly higher than 31.8% and 11.8% in adult patients with cancer (28). This difference may be due to the differences in tumor types, chemotherapeutic drugs, and the potential disease status of the study population. Another reason may be that 84.04% (237/282) of the children received intra-venous and or intra-arterial chemotherapy within 6 months before this investigation. For IVC, our institution adopts the standard protocol, i.e., intravenous vincristine, etoposide, and carboplatin for 6 cycles, once every 3 to 4 weeks. For IAC, the chemotherapy drugs are melphalan + topotecan and melphalan + carboplatin. For IAC, there was no strong evidence showing that chemotherapy drugs affect anemia or bone marrow suppression. However, compared to IVC, the occurrence of anemia and bone marrow suppression after IAC was greatly reduced, and anemia and bone marrow suppression were transient, causing less damage to children with Rb (29).

The causes of anemia and bone marrow suppression in many patients with cancer are unclear (30). In a retrospective study, malnutrition, >3 previous chemotherapies, and a combination of more than three drugs were significantly associated with severe bone marrow suppression (31). In the child model, lymphopenia was the strongest predictor of the risk model of severe anemia, followed by Hb levels and performance status (32). Variables identified as predictors of anemia were associated with patient characteristics: baseline Hb <13.5 g/dL, age >60 years, and body mass index <25 kg/m^2^ (33). In the univariate and multivariate regression analysis and predictive model generated by the multivariate regression model, we found that many factors can affect anemia and bone marrow suppression after IAC. The anemia severity and bone marrow suppression severity before IAC had a significant effect on anemia severity, and as the grade increased, its effect on post-IAC anemia was stronger. The reason for this situation may be that “bone marrow suppression” was comprehensively assessed by WBC, Hb, and PLT levels, which can objectively show the patient’s blood status before IAC. Regarding the “degree of bone marrow suppression,” variables such as IVC within 1–2 months before IAC, red cell volume distribution width, and serum direct bilirubin affect the degree of bone marrow suppression. Possible causes may need to be further explored.

This study had some limitations. First, this study is a retrospective study, and in the 282 children, the routine blood tests of some children were incomplete, and the rate of missing values of laboratory information reached 19.5%. Although we excluded patients with missing data in the univariate and multivariate regression analyses, further high-quality evidence is necessary to guide clinical practice. Second, the interval of routine blood examination before and after IAC was inconsistent. Some children with Rb who had a long time interval related to routine blood tests may have experienced factors with a probable effect on their results, such as an increased WBC level caused by infection. Finally, our research only recorded the results of routine blood tests after a single session of IAC and failed to reflect the dynamic changes in serological indicators caused by IAC. However, the large sample size can compensate for these limitations to a certain extent, and there was no statistically significant deviation in the conclusion that “IAC will increase the risk of anemia and bone marrow suppression in children with Rb.” In subsequent research, we will increase the frequency of routine blood tests in children with Rb in each period after IAC and include other serological indicators, such as liver and kidney function and coagulation function to determine the factors that guide the prediction of dynamic changes in serology and adverse reactions after IAC and improve the management of children with Rb.

## Conclusion

Children with Rb have an increased risk of anemia and myelosuppression after IAC, but this is temporary; therefore, IAC should be considered as the standard of care. The univariate and multivariate regression model results showed several factors that may affect the occurrence of anemia and bone marrow suppression after IAC. Based on the model, we generated equations to calculate the anemia severity and degree of bone marrow suppression, which played a clinical guiding role in the prediction and timely control of anemia and bone marrow suppression after IAC. The model was developed using data of patients with retinoblastoma and needs to be validated in a larger population.

## Data Availability Statement

The original contributions presented in the study are included in the article/**Supplementary Material**. Further inquiries can be directed to the corresponding authors.

## Ethics Statement

This study has been approved by the Ninth people’s hospital, Shanghai Jiao Tong University School of Medicine (SH9H-2019-T291-3). Written informed consent from the participants’ legal guardian/next of kin was not required to participate in this study in accordance with the national legislation and the institutional requirements.

## Author Contributions

CZ wrote the manuscript as the first author. Professor LH, XS, XW, and LL conceived and designed the study. MH and JF contributed to the data analysis, data interpretation, and manuscript revision. XH and RJ were involved in the data collection. All authors read and approved the final manuscript.

## Funding

This study was supported by the Gaoyuan Nursing Grant Support of Shanghai Municipal Education (Hlgy1808kyx); the Shanghai Jiao Tong University School of Medicine: Nursing Development Program; the Nursing Program of Shanghai Ninth People’s Hospital, Shanghai Jiao Tong University School of Medicine (JYHL20193D05). The above-mentioned funding sponsor played no role in the study design; data collection, analysis, and interpretation; and manuscript writing. Innovative research team of high-level local universities in Shanghai (SHSMU- ZDCX20210902); Clinical Research Plan of SHDC (SHDC2020CR1009A).

## Conflict of Interest

The authors declare that the research was conducted in the absence of any commercial or financial relationships that could be construed as a potential conflict of interest.

## Publisher’s Note

All claims expressed in this article are solely those of the authors and do not necessarily represent those of their affiliated organizations, or those of the publisher, the editors and the reviewers. Any product that may be evaluated in this article, or claim that may be made by its manufacturer, is not guaranteed or endorsed by the publisher.
