# Supplementary material for: Anemia and Bone Marrow Suppression After Intra-Arterial Chemotherapy in Children With Retinoblastoma: A Retrospective Analysis
Source: Front Oncol. 2022 Jul 25;12:848877. doi: 10.3389/fonc.2022.848877 (PMC9359439; doi:10.3389/fonc.2022.848877)
Supplement: Supplementary file 1 [file Table_1.docx]

**Supplementary files**

STable 1. Univariate regression analysis of IAC according to anemia severity and degree of bone marrow suppression.

|  | **Total patients (N = 282)** | | | | | | | | | | | | |
| --- | --- | --- | --- | --- | --- | --- | --- | --- | --- | --- | --- | --- | --- |
|  | **Anemia (N = 194)** | | | | | |  | **Bone marrow suppression (N = 197)** | | | | | |
|  | **Worse** |  | **Unchanged** |  | **Better** | **p value^#^** |  | **Worse** |  | **Unchanged** |  | **Better** | **p value^#^** |
| **Sex** |  |  |  |  |  | 0.559 |  |  |  |  |  |  | 0.536 |
| Male | 45 (56.2) |  | 56 (60.2) |  | 10 (47.6) |  |  | 35 (54.7) |  | 55 (56.1) |  | 23 (65.7) |  |
| Female | 35 (43.8) |  | 37 (39.8) |  | 11 (52.4) |  |  | 29 (45.3) |  | 43 (43.9) |  | 12 (34.3) |  |
| **Age (mean (SD))^&^** | 3.95 (1.35) |  | 3.69 (1.29) |  | 3.66 (1.08) | 0.384 |  | 3.93 (1.17) |  | 3.71 (1.22) |  | 3.71 (1.76) | 0.553 |
| **Times of IAC** | |  |  |  |  | 0.1922 |  |  |  |  |  |  | 0.2686 |
| 1 | 20 (25.0) |  | 26 (28.0) |  | 9 (42.9) |  |  | 14 (21.9) |  | 8 (22.9) |  | 34 (34.7) |  |
| 2 | 23 (28.7) |  | 23 (24.7) |  | 6 (28.6) |  |  | 15 (23.4) |  | 8 (22.9) |  | 30 (30.6) |  |
| 3 | 19 (23.8) |  | 26 (28.0) |  | 1 (4.8) |  |  | 20 (31.2) |  | 10 (28.6) |  | 17 (17.3) |  |
| 4 | 9 (11.2) |  | 11 (11.8) |  | 4 (19.0) |  |  | 10 (15.6) |  | 6 (17.1) |  | 8 (8.2) |  |
| ≥5 | 9 (11.2) |  | 7 (7.6) |  | 1 (4.8) |  |  | 5 (7.8) |  | 3 (8.6) |  | 9 (9.1) |  |
| **Chemotherapy drugs** | |  |  |  |  | 0.561 |  |  |  |  |  |  | 0.884 |
| Melphalan + Topotecan | 49 (61.3) |  | 50 (53.8) |  | 13 (61.9) |  |  | 38 (59.4) |  | 57 (58.2) |  | 19 (54.3) |  |
| Melphalan + carboplatin | 31 (38.8) |  | 43 (46.2) |  | 8 (38.1) |  |  | 26 (40.6) |  | 41 (41.8) |  | 16 (45.7) |  |
| **Duration of surgery (min) (mean (SD)) ^&^** | 69.59 (16.53) |  | 71.03 (22.94) |  | 67.86 (25.62) | 0.789 |  | 73.98 (22.35) |  | 69.06 (20.02) |  | 65.31 (18.21) | 0.111 |
| **Durartion of heparin applied (min) (mean (SD)) ^&^** | 65.55 (13.83) |  | 65.83 (19.87) |  | 64.52 (23.38) | 0.956 |  | 69.22 (19.09) |  | 64.76 (18.10) |  | 60.89 (13.67) | 0.072 |
| **Height (cm) (mean (SD)) ^&^** | 90.69 (11.04) |  | 87.69 (11.80) |  | 89.29 (10.94) | 0.232 |  | 90.04 (10.41) |  | 88.04 (10.08) |  | 89.68 (16.45) | 0.524 |
| **Weight (kg) (mean (SD)) ^&^** | 13.79 (2.95) |  | 12.87 (3.31) |  | 13.24 (2.80) | 0.163 |  | 13.37 (2.93) |  | 13.07 (2.77) |  | 13.55 (4.37) | 0.697 |
| **BMI (mean (SD)) ^&^** | 16.71 (1.64) |  | 16.59 (1.59) |  | 16.59 (1.84) | 0.876 |  | 16.38 (1.50) |  | 16.78 (1.69) |  | 16.66 (1.79) | 0.338 |
| **Head circumference (cm) (mean (SD)) ^&^** | 47.66 (2.48) |  | 47.37 (2.79) |  | 47.91 (2.86) | 0.681 |  | 47.44 (2.46) |  | 47.44 (2.64) |  | 47.72 (3.52) | 0.883 |
| **Chest circumference (cm) (mean (SD)) ^&^** | 52.12 (4.12) |  | 50.94 (4.31) |  | 52.56 (3.97) | 0.145 |  | 51.54 (3.59) |  | 51.12 (4.14) |  | 52.83 (5.38) | 0.166 |
| **Operating doctors** | |  |  |  |  | 0.752 |  |  |  |  |  |  | 0.684 |
| Dr. one | 58 (72.5) |  | 72 (77.4) |  | 16 (76.2) |  |  | 50 (78.1) |  | 71 (72.4) |  | 27 (77.1) |  |
| Dr. two | 22 (27.5) |  | 21 (22.6) |  | 5 (23.8) |  |  | 14 (21.9) |  | 27 (27.6) |  | 8 (22.9) |  |
| **Duration of Rb (mean (SD)) ^&^** | 10.01 (12.08) |  | 8.43 (9.18) |  | 8.40 (6.96) | 0.573 |  | 8.81 (8.85) |  | 9.36 (11.78) |  | 8.21 (7.84) | 0.842 |
| **Intravenous chemotherapy within 1 months before IAC** | | | | |  | 0.056 |  |  |  |  |  |  | 0.162 |
| Yes | 77 (96.2) |  | 83 (89.2) |  | 17 (81.0) |  |  | 62 (96.9) |  | 87 (88.8) |  | 31 (88.6) |  |
| None | 3 (3.8) |  | 10 (10.8) |  | 4 (19.0) |  |  | 2 (3.1) |  | 11 (11.2) |  | 4 (11.4) |  |
| **Arterial chemotherapy within 1 months before IAC** | | | | |  | 0.5 |  |  |  |  |  |  | 0.496 |
| Yes | 28 (35.0) |  | 28 (30.1) |  | 9 (42.9) |  |  | 22 (34.4) |  | 36 (36.7) |  | 9 (25.7) |  |
| None | 52 (65.0) |  | 65 (69.9) |  | 12 (57.1) |  |  | 42 (65.6) |  | 62 (63.3) |  | 26 (74.3) |  |
| **Intravenous chemotherapy within 3 months before IAC** | | | | |  | 0.042 |  |  |  |  |  |  | 0.149 |
| Yes | 64 (80.0) |  | 61 (65.6) |  | 12 (57.1) |  |  | 51 (79.7) |  | 67 (68.4) |  | 22 (62.9) |  |
| None | 16 (20.0) |  | 32 (34.4) |  | 9 (42.9) |  |  | 13 (20.3) |  | 31 (31.6) |  | 13 (37.1) |  |
| **Arterial chemotherapy within 3 months before IAC** | | | | |  | 0.798 |  |  |  |  |  |  | 0.705 |
| Yes | 21 (26.2) |  | 27 (29.0) |  | 7 (33.3) |  |  | 17 (26.6) |  | 31 (31.6) |  | 9 (25.7) |  |
| None | 59 (73.8) |  | 66 (71.0) |  | 14 (66.7) |  |  | 47 (73.4) |  | 67 (68.4) |  | 26 (74.3) |  |
| **Intravenous chemotherapy within 6 months before IAC** | | | | |  | 0.006 |  |  |  |  |  |  | 0.172 |
| Yes | 63 (78.8) |  | 56 (60.2) |  | 10 (47.6) |  |  | 48 (75.0) |  | 64 (65.3) |  | 20 (57.1) |  |
| None | 17 (21.2) |  | 37 (39.8) |  | 11 (52.4) |  |  | 16 (25.0) |  | 34 (34.7) |  | 15 (42.9) |  |
| **Arterial chemotherapy within 6 months before IAC** | | | | |  | 0.638 |  |  |  |  |  |  | 0.59 |
| Yes | 19 (23.8) |  | 26 (28.0) |  | 7 (33.3) |  |  | 16 (25.0) |  | 30 (30.6) |  | 8 (22.9) |  |
| None | 61 (76.2) |  | 67 (72.0) |  | 14 (66.7) |  |  | 48 (75.0) |  | 68 (69.4) |  | 27 (77.1) |  |
| **Serum creatinine (median [IQR]) ^&^** | 23.00 [19.00, 26.98] |  | 27.00 [24.72, 31.00] |  | 26.00 [24.00, 32.70] | <0.001 |  | 24.00 [19.06, 28.38] |  | 26.00 [23.00, 30.98] |  | 26.50 [22.00, 29.75] | 0.09 |
| **Bile acids (median [IQR]) ^&^** | 4.50 [2.90, 7.00] |  | 3.17 [1.90, 6.75] |  | 2.88 [1.55, 4.25] | 0.046 |  | 4.15 [2.56, 7.10] |  | 3.60 [2.20, 6.80] |  | 3.10 [2.45, 6.30] | 0.633 |

#The p value was calculated by the chi-square test.

&The p value was calculated by the t test or Mann-Whitney test
